# Effect of Insect Live Larvae as Environmental Enrichment on Poultry Gut Health: Gut Mucin Composition, Microbiota and Local Immune Response Evaluation

**DOI:** 10.3390/ani11102819

**Published:** 2021-09-27

**Authors:** Elena Colombino, Ilaria Biasato, Ilario Ferrocino, Sara Bellezza Oddon, Christian Caimi, Marta Gariglio, Sihem Dabbou, Marta Caramori, Elena Battisti, Stefania Zanet, Ezio Ferroglio, Luca Cocolin, Laura Gasco, Achille Schiavone, Maria Teresa Capucchio

**Affiliations:** 1 Department of Veterinary Sciences, University of Turin, 10095 Grugliasco, TO, Italy; marta.gariglio@unito.it (M.G.); marta.caramori@edu.unito.it (M.C.); elena.battisti@unito.it (E.B.); stefania.zanet@unito.it (S.Z.); ezio.ferroglio@unito.it (E.F.); achille.schiavone@unito.it (A.S.); mariateresa.capucchio@unito.it (M.T.C.); 2 Department of Agricultural, Forestry and Food Sciences, University of Turin, 10095 Grugliasco, TO, Italy; ilaria.biasato@unito.it (I.B.); ilario.ferrocino@unito.it (I.F.); sara.bellezzaoddon@unito.it (S.B.O.); christian.caimi@unito.it (C.C.); lucasimone.cocolin@unito.it (L.C.); laura.gasco@unito.it (L.G.); 3 Center Agriculture Food Environment (C3A), University of Trento, 38010 San Michele all’Adige, TN, Italy; sihem.dabbou@unitn.it; 4 Institute of Sciences of Food Production, CNR, 10095 Grugliasco, TO, Italy

**Keywords:** poultry, insect, *Hermetia illucens*, *Tenebrio molitor*, gut health, mucin, cytokines, microbiota

## Abstract

**Simple Summary:**

*Hermetia illucens* (HI) and *Tenebrio* *molitor* (TM) are considered innovative protein sources in animal nutrition. Recently, it has been discovered that low doses of insect meal can also act as gut prebiotics, providing an alternative to antibiotics. This is extremely relevant to the enhancement of gut health, specifically the absence, avoidance and prevention of gastrointestinal diseases in order to maintain animal welfare, health and performance. Insect meals have been proven to be effective in modulating gut morphometry, mucin composition and microbiota. However, no studies are available on the effects of live insect larvae. For these reasons, this study describes for the first time the effects of low doses of HI and TM live larvae as environmental enrichment on chicken mucin expression, local immune response and microbiota composition. The results herein obtained demonstrated that live insect larvae administered based on 5% of the expected daily feed intake did not impair mucin expression or local cytokine production. Furthermore, it seemed to produce a slight improvement of the caecal microbiota by enhancing a minor fraction of potentially beneficial taxa able to produce short chain fatty acids as an important source of energy for the enterocytes.

**Abstract:**

The aim of this study was to evaluate the effect of *Hermetia illucens* (HI) and *Tenebrio molitor* (TM) live larvae as environmental enrichment on the mucin composition, local immune response and microbiota of broilers. A total of 180 four-day-old male broiler chickens (Ross 308) were randomly allotted to three dietary treatments (six replicates/treatment; ten animals/replicate): (i) control (C); (ii) C+HI; (iii) C+TM. Live larvae were distributed based on 5% of the expected daily feed intake. At slaughter (39 days of age), samples of duodenum, jejunum and ileum (twelve animals/diet) were submitted to mucin histochemical evaluation. Expression of MUC-2 and cytokines was evaluated by rt-qPCR in jejunum. Mucin staining intensity was not influenced by diet (*p* > 0.05); however, this varied depending on the intestinal segment (*p* < 0.001). No significant differences were recorded for IL-4, IL-6 TNF-α, MUC-2 and INF-γ gene expression in jejunum, while IL-2 was lower in the TM group compared to HI and C (*p* = 0.044). Caecal microbiota showed higher abundance of *Clostridium, Saccharibacteria* and *Victivallaceae* in the HI group, while *Collinsella* was higher in the TM group. The results suggested that live insect larvae did not impair mucin composition or local immune response, and can slightly improve caecal microbiota by enhancing a minor fraction of short chain fatty acid-producing taxa.

## 1. Introduction

Insects are considered a valuable and innovative alternative to fish and soybean meals in animal nutrition due to their high content of high-quality protein, fat, minerals, vitamins and fibre [1]. In particular, the most promising species are represented by the black soldier fly (*Hermetia illucens*, HI) and the yellow mealworm (*Tenebrio molitor*, TM), thanks to their easy rearing systems and their ability to consume a wide range of organic side streams [2].

In poultry, insects have been mainly administered as full-fat or defatted insect meal to substitute for traditional protein sources, but they can also be provided as live larvae for environmental enrichment [3,4]. In fact, insects are part of the natural diet of chickens, which are highly motivated to interact with them and consume them [4]. Thus, the administration of insect live larvae can increase broilers’ foraging behaviour and activity, improving chicken leg health and welfare [4,5]. Furthermore, insects attract increasing interest as their exoskeleton is rich in chitin, an indigestible polysaccharide composed of N-acetylglucosamine residues which are linked by β-(1,4)-glycosidic bonds [6]. Previous research has suggested that this bioactive compound can have antimicrobial and immunostimulant effects, showing that it can act as an alternative to antibiotics and be beneficial to gut health [7].

Gut health is defined as the absence, prevention and avoidance of intestinal disease so that animal health, welfare and performances are maintained [8]. The key factors for guaranteeing an optimal gut health appear to be gut mucin expression, local immune response and gut microbiota, which together contribute to the “intestinal immunity” [9]. Firstly, the mucus layer that covers the gut mucosa contains mucins secreted by goblet cells; it represents the “chemical barrier” that regulates contact between the commensal bacteria and the epithelial cells [9]. Secondly, the gut immune system forms the “immunological barrier” where macrophages and dendritic cells present antigens to T cells, resulting in the differentiation and activation of various T cell subsets, secretion of IgA, chemokines and cytokines [9]. Thirdly, gut microbiota indicates all of the microorganisms inhabiting the gastrointestinal tract, and constitutes the “microbiological barrier” which provides colonization resistance against pathogens, produces metabolites that modulate immune signalling, and promotes immune homeostasis [10]. Previous studies have demonstrated that all these components can be driven by dietary treatments [11,12,13].

Particularly, insect meals have been reported to be able to positively modulate gut health in pigs [14], poultry [11], ducks [15] and aquaculture [16]. In poultry, the effects of HI and TM insect meals on gut morphometry, mucin composition and microbiota have been already investigated, showing promising results [17,18]. However, little is known about the effects on chicken gut health of insect live larvae administered as environmental enrichment. To our best knowledge, only Bellezza-Oddon et al. [19] have evaluated the effect of HI and TM live larvae on broiler gut morphometry, while no studies are currently available on mucin composition, local immune response or gut microbiota. Therefore, the present study aimed to evaluate the effect of HI and TM live larvae as environmental enrichment on the mucin composition, local immune response and microbiota of broiler chickens.

## 2. Materials and Methods

### 2.1. Birds, Diet and Chitin Determination

The present experiment was conducted at the poultry facility of the University of Turin (Italy). The experimental protocol (ID: 814715) was approved by the Ethical Committee of the University of Turin (Italy). The experimental design of the present study was reported in Bellezza-Oddon et al. [19]. Briefly, a total of 180 four-day-old male broiler chickens (Ross 308) were randomly allotted to three dietary treatments (6 replicates/treatment; 10 animals/replicate): (i) C: commercial diet (Famaarco SPA, Cuneo, Italy); (ii) HI: C + HI live larvae; (iii) TM: C + TM live larvae. The commercial diet was based on soybean meal, corn and soybean oil with the addition of a vitamin–mineral premix, synthetic aminoacids, cocciodiostatics and histomonostatics (Famaarco SPA, Cuneo, Italy). The nutrient composition of the two diets (starter: 1–11 day and grower-finisher: 12–38 day) is reported in Table 1. Live larvae were distributed in two plates once a day (11:00 am) based on 5% of the expected daily feed intake, using two different sizes of larvae for starter (length: 0.80 cm) and grower (length: 1.5 cm) periods. To avoid potential bias, two plates with control feed were also provided once a day (11:00 am) to the C animals.

The chitin content of the whole larvae was analysed following the method described by Woods et al. [20]. The samples were totally defatted by solvent extraction, and subsequently subjected to demineralisation and deproteinization. All the values are reported as means of quadruplicate analyses.

At 39 days of age, 18 birds/treatment (3 birds/replicate) were electrically stunned and slaughtered.

### 2.2. Mucin Histochemical Staining and Quantification

Twelve birds per feeding group were submitted to histochemical investigations. At slaughter, samples of duodenum (loop of the duodenum), jejunum (tract before Meckel’s diverticulum) and ileum (tract before ileo-colic junction) were excised, flushed with 0.9% saline to remove all the content and fixed in 10% buffered formalin solution. Tissues were routinely embedded in paraffin wax blocks, sectioned at 5 μm thickness and mounted on glass slides. The paraffin-embedded intestinal sections (duodenum, jejunum and ileum) were submitted to a triple histochemical staining that demonstrated the three different mucin subtypes.

Neutral mucins were stained magenta through the periodic acid–Schiff (PAS) staining according to Forder et al. [21].

Acidic sialylated mucins were identified in blue by Alcian Blue, pH 2.5 (AB) staining. Sections were brought to water, immersed in 8 G X Alcian blue in 3% acetic acid solution for 30 min, washed in running tap water for 5 min, dehydrated and mounted on glass slides [22].

Acidic sulfated mucins were identified in purple-black by high iron diamine (HID) staining. Sections were brought to water, oxidized in 1% periodic acid solution for 10 min and washed in running tap water for 5 min. Sections were then immersed in the HID solution (120 mg metadiamine, 20 mg paradiamine and 1.4 mL 10% ferric chloride in 50 ml of distilled water) for 18 h, rinsed in tap water, dehydrated and mounted [22].

One slide per histochemical staining for each intestinal segment was examined by light microscopy. Five randomly selected high-power fields per slide were captured with a Nikon DS-Fi1 digital camera coupled to a Zeiss Axiophot microscope using a 20× objective lens. NIS-Elements F software was used for image capturing. Mucin staining quantification was then performed by Image^®^-Pro Plus software by means of pixels classification. Mucin quantification was expressed as the percentage of the gut mucosal area (covering both the crypts and the villi) that was positive for the evaluated histochemical staining [23]. Total mucin content was obtained by adding the PAS, AB and HID percentage of each intestinal segment.

### 2.3. Real Time Quantitative PCR (rt-qPCR)

At slaughter, jejunum from 12 birds/treatment was aseptically collected, placed 24 h in RNAlater (Sigma-Aldrich, MO, USA) at 4 °C and then stored at −80 °C. Total RNA was then extracted using TRIzol reagent (Invitrogen, Carlsbad, CA, USA) in accordance with manufacturer’s instructions. The RNA quality of every sample was quantified by Nanodrop 1000 spectrophotometer (Thermo Fisher Scientific Inc., Wiington, DE, USA) and the ratio (OD260:OD280) ranged from 1.7 to 2.1. Afterwards, 2.0 μg of total RNA for each sample was reverse transcribed to cDNA by using the iScript™ cDNA Synthesis Kit (Bio-Rad Laboratories, Inc., Hercules, CA, USA) according to manufacturer protocol, and the cDNA was stored at −20 °C. rt-qPCR was performed using a 7500 Real Time PCR system (Applied Biosystems, Waltham, MA) in a 20 μL reaction mixture containing 2 μL cDNA, 10 μL of SYBR Green Supermix kit (Bio-Rad Laboratories, Inc., Hercules, CA, USA) and 0.1 μL of forward and reverse primers (40 mM) of the selected genes. Primers used for selected genes (IL-2, IL-4, IL-6 TNF-α, INF-γ and MUC-2) were designed based on the available sequences in GenBank and synthetized by Macrogen Inc. (Amsterdam, the Netherlands) (Table 2). Thermal conditions for performing rt-qPCR were as follow: initial incubation at 95 °C for 30 s; 40 cycles of denaturation at 95 °C for 15 s and annealing/extension at 60 °C for 60 s following by a melt curve analysis (65–95 °C with 0.5 °C increments at 2–5 s/step). Relative standard curve method was performed using B-actin and GAPDH as internal control genes to normalize for RNA abundance. Each reaction was run in triplicate. Efficiency curves were performed for each primer set using log10 diluted cDNA in order to obtain efficiency-corrected relative quantification. Amplification efficiency between 90 and 110% was considered good with correlation coefficient (R^2^) of 0.99 [24].

### 2.4. Caecal Microbiota Characterization

At slaughter, samples of caecal content were collected from 18 birds/treatment (3 birds/replicate) and stored at −80 °C prior to DNA extraction and sequencing.

Total DNA was extracted from each sample using the RNeasy Power Microbiome KIT (Qiagen. Milan. Italy) following the manufacturer’s instructions. One microliter of RNase (Illumina Inc. San Diego. CA) was added to digest RNA in the DNA samples with an incubation of 1 h at 37 °C. DNA was quantified using Nanodrop 1000 spectrophotometer (Thermo Fisher Scientific Inc., Wiington, DE, USA) and standardized at 5 ng/μL.

The extracted DNA was used to assess the microbiota by the amplification of the V3-V4 region of the 16S rRNA gene (F: 5′-TCGTCGGCAGCGTCAGATGTGTATAAGAGA CAGCCTACGGGNGGCWGCAG-3′; R: 5′-GTCTCGTGGGCTCGGAGATGTGTATAA GAGACAGGACTACHVGGGTATCTAATCC-3′) [25]. The PCR products were purified according to the Illumina metagenomic standard procedure (Illumina Inc., San Diego, CA, USA). Sequencing was performed with a MiSeq Illumina instrument with V3 chemistry and generated 250 bp paired-end reads in accordance with the manufacturer’s instructions.

### 2.5. Bioinformatics and Statistical Analysis

Statistical analysis was conducted using R software version 4.0.4 (R Foundation for Statistical Computing, Vienna, Austria; http://www.r-project.org. The Shapiro–Wilk test was used to test the normality of the data distribution.

Data regarding mucin staining intensity were analysed by a robust two-way ANOVA test (trimmed means method) using the “walrus” R package. The two-way ANOVA test allowed the evaluated variables to depend on three fixed factors (diet, intestinal segment, and the interaction between them). The interactions were evaluated by robust pairwise comparisons.

For rt-qPCR, Microsoft Excel was used to convert the quantification cycle (Cq) values to linear units called relative normalized expression in accordance with Taylor et al. [26]. Briefly, the average Cq of all samples in the control group for each target was determined and the relative difference (DCq) with the mean Cq per individual sample for each target gene was assessed. The relative quantities were calculated according to reaction efficiency (efficiency^DCq). For each sample, a normalization factor was determined from the geometric mean of the associated reference gene relative quantities. The relative normalized expression for each target gene was then calculated per sample by dividing the relative quantity by the normalization factor. Samples with relative normalized expression >10 were identified as potential outliers and excluded from the analysis. A robust one-way ANOVA test was performed, followed by robust pairwise comparisons.

Data were described as mean and standard deviation (SD). *p* values < 0.05 were considered statistically significant. R scripts are provided in the Supplementary Materials.

Regarding microbiota, paired-end reads were first merged using FLASH software with default parameters [27]. Joint reads were further quality filtered (at Phred < Q20) using QIIME 1.9.0 software through a multiple_split_libraries_fastq.py script [28] and the pipeline recently described in [29]. OTU clustering was obtained at 97% of similarity by the pick_otus.py script and taxonomy assignment was assessed by Greengenes 16S rRNA gene database v. 2013 using the RDP Classifier, with a minimum confidence score of 0.80. The OTU table was rarefied at the lowest number of sequence (12.337 reads) and display the higher taxonomy resolution. The vegan package of R was used to calculate the alpha diversity [30]. The diversity indices were further analysed using the Wilcoxon rank sum test to assess differences between the dietary treatments. Weighted UniFrac distance matrices and OTU table generated through QIIME were used to perform Adonis and Anosim statistical tests in R environment. Scripts are provided in the Supplementary Materials. A Generalized Linear Model was used in order to test the importance of insect administration on the relative abundance of OTU.

## 3. Results

### 3.1. Birds, Diet and Chitin Determination

The chitin content of HI and TM whole larvae was determined to be 8.84% and 5.13% of dry matter, respectively.

### 3.2. Mucin Histochemical Evaluation

Data regarding mucin staining intensity in the gut of the evaluated chickens are reported in Table 3. No significant differences were recorded for neutral mucins, sialomucins, sulfomucins, and total mucins among the three dietary treatments (*p* > 0.05). On the contrary, significant differences were recorded for all of the evaluated mucins among the intestinal segments, showing a proximo-distal increasing gradient from duodenum to ileum (*p* < 0.001).

### 3.3. Real Time Quantitative PCR (rt-qPCR)

Gene expression in the jejunum of the broiler chickens are summarised in Table 4. IL-2 expression was influenced by diet, being lower in the TM group when compared to the other groups (*p* = 0.044). The other evaluated cytokines and MUC-2 expression were not influenced by diet (*p* > 0.05).

### 3.4. Caecal Microbiota Characterization

**Figure 1:**
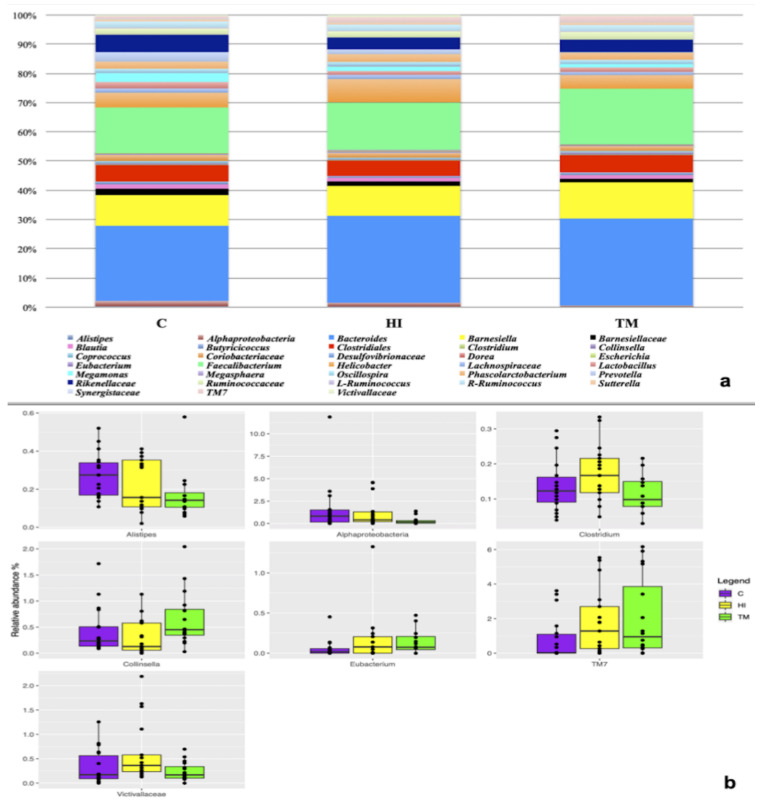
(**a**) Composition of the caeca microbiota in the three dietary treatments (C: control; HI: *Hermetia illucens*; TM: *Tenebrio molitor*). (**b**) Differentially abundant OTU as a function of the dietary treatments. (C: control; HI: *Hermetia illucens*; TM: *Tenebrio molitor*).

At the end of the trial, 54 caecal samples were sequenced. After sequencing, 1,849,109 reads were obtained and after the quality filtering, 995,342 reads were used for the downstream analysis with an average value of 18,432 ± 6093 reads/sample. No differences in alpha or beta diversity were observed among the three dietary treatments (*p* > 0.05). The microbiota in the three diets was characterized by the presence of Rikenellaceae and Ruminococcaceae families. At genus level, *Bacteroides*, *Faecalibacterium*, *Barnesiella*, *Helicobacter* and *Phascolarctobacterium* were the most abundant (Figure 1a). However, it was possible to observe that the minor OTU fraction (relative abundance < 5%) was influenced by HI and TM live larvae inclusion (*p* < 0.05). In detail, HI inclusion was characterized by the presence of *Clostridium*, *Saccharibacteria* (TM7) and Victivallaceae, while TM showed a higher abundance of *Collinsella*. On the other hand, *Eubacterium* was enriched in both HI and TM groups compared to C (Figure 1b).

## 4. Discussion

*Hermetia illucens* and *Tenebrio molitor* meals have been widely studied as innovative protein sources for poultry nutrition [31]. Recently, it has been discovered that insect live larvae can be used as a valuable environmental enrichment for poultry [4]. Also, low doses of insect meal can act as gut probiotics, being an alternative to antibiotics [7]. These aspects are particularly relevant considering consumer pressure and worries toward animal welfare and the harmful effects of antibiotics [5,32].

Thus, this study describes for the first time the effects of low doses of HI and TM live larvae as environmental enrichment on chicken mucin dynamics, local immune response and microbiota composition.

Regarding mucin composition, no significant differences were recorded among the three dietary treatments for neutral mucins, sialomucins, sulfomucins, or total mucins (*p* > 0.05). Furthermore, MUC-2 gene expression was comparable in the C, HI and TM groups (*p* > 0.05). Mucin 2 (MUC-2) is the main type of secretory mucin in the intestine; it constitutes the mucus layer that covers the epithelium. As a physical–chemical barrier to prevent microbes from direct contact with epithelial cells, this mucus layer controls microflora colonization by providing colonization sites and entrapping invasive bacteria [33]. The obtained results are in contrast with previous studies using HI and TM meal inclusion in broiler diet. In fact, low levels of HI and TM meal (5%) showed higher mucin staining intensity in the intestinal villi when compared to high levels [11,17,18]. On the other hand, higher levels of HI and TM meals (10–15%) had a detrimental effect, with a significant reduction of villi mucin staining intensity [11,17,18]. The lack of effects observed in the present study could be attributed to the different form in which insects were provided to the animals. Defatted or full-fat insect meals are obtained through a heating-drying process in order to concentrate nutrients, while insect live larvae are richer in water [34]. For this reason, the fresh whole insect dose needs to be doubled compared to insect meal to reaching the same nutrient concentration [34]. In this study, insects were administered based on 5% of the expected daily feed intake, which is far less than the 5% of TM or HI meal used in the above-mentioned studies [23,35]. Irrespective of diet, all the evaluated mucins showed a proximo-distal increasing gradient, being greater in the ileum and jejunum when compared to the duodenum (*p* < 0.001). This is in accordance with the physiological development of the chicken gastrointestinal tract and has been also reported to be maintained in previous studies using HI and TM meals in poultry nutrition [11,17,21,29,35]. In particular, Forder et al. [21] observed an increase in the density of goblet cells along the duodenal-ileal axis, suggesting that the distal ileum may be a preferred region for bacterial colonization.

Considering local immune response, the expression of intestinal cytokines was not influenced towards “pro” or “anti” inflammatory patterns by HI and TM live larvae feeding. IL-2 was down-regulated in the jejunum of the TM group (*p* = 0.044). IL-2 is considered a pro-inflammatory cytokine along with IFN-γ and TNF-α, and it is involved in a wide range of signalling events within cells such as necrosis or apoptosis, amplification of antigen presentation, increased production of Reactive Oxygen Species and T-cell proliferation/differentiation [36,37,38]. On the contrary, anti-inflammatory cytokines include IL-4 and IL-6; they are immunoregulatory molecules that control the pro-inflammatory cytokines’ response [39]. No previous studies are available on gut mucosal immune system regulation after HI/TM meal or live larvae administration in chickens. However, a recent study [14] evaluated the effect of HI meal inclusion (4.0% and 8.0%) on pig mucosal immune status, showing a down-regulation of pro-inflammatory cytokines and an up-regulation of anti-inflammatory cytokines. Therefore, the reduction of IL-2 in the duodenum of TM chickens is in accordance with Yu et al. [14] and should be considered a positive change that benefits mucosal homeostasis, helping to avoid aberrant immune response. This can be attributed to the lower chitin content of TM larvae registered in the present study compared to HI larvae, which is in accordance with the recent literature [40,41,42,43,44]. Chitin has been reported to have the ability to induce cytokine production [45]. For this reason, the lower concentration of this active biocompound in TM larvae could be responsible for the lower transcription levels of IL-2. This result seems to further support the immunoregulatory properties attributed to chitin and chitosan, even if further studies will be required to clarify the underlying mechanisms [14].

Finally, both mucin composition and gut immune response can be influenced by shifts in the microbiota [46,47]. In the present study, the composition of the microbiota of caecal samples was characterized by a high presence of *Bacteroides, Faecalibacterium, Barnesiella, Helicobacter* and *Phascolarctobacterium*, in accordance with Qi et al. [48] and Clavijo et al. [49]. Among the reported bacterial genera, *Helicobacter* can play a positive role in the cecum because some species produce the enzyme hydrogenase, which seems to stimulate the production of short chain fatty acids (SCFAs) [49]. However, *Helicobacter* deserves attention as little is known about the pathogenic and zoonotic potential of this species, which can colonize the chicken gut [50]. Moreover, *Bacteroides* are considered to have one of the highest hydrolytic activities among all known genera, being recognized as effective degraders of non-digestible carbohydrates and SCFA producers [51]. As is known, SCFAs have positive health effects, especially butyric acid, which has been shown to have anti-inflammatory properties, to modulate oxidative stress and to be a main energy substrate for enterocytes [52].

Furthermore, HI and TM live larvae influenced the minor OTU fraction, the Victivillaceae family, *Saccharibacteria* (TM7) and *Clostridium* genus being higher in HI group than in the others. No previous studies in poultry reported the presence of the Victivillaceae family, but it has been isolated in human caeca, encompassing a number of anaerobic, cellobiose-degrading bacteria that are positively involved in polysaccharide fermentation [53]. To date, *Saccharibacteria* has previously been detected in ducks but its role in poultry microbiota remains poorly understood [54]. *Clostridium* represents one of the main bacterial genera observed in the chicken cecum, and it also encompasses bacteria capable of producing butyric acid, which has been reported to positively influence intestinal villus structure and control naturally occurring pathogens, as well as to show remarkable anti-inflammatory properties [55]. Moreover, *Collinsella* was more abundant in the TM group when compared to the other diets. *Collinsella* seems to be able to affect host lipid metabolism through altering intestinal cholesterol absorption, decreasing glycogenesis in the liver and increasing triglyceride synthesis [56]. In both the HI and the TM groups an increase in *Eubacterium* was also detected; the presence of this taxa can exert a beneficial effect on gut mucosa as it may contribute to the hydrolysis of starch and other macromolecules, with the subsequent formation of SCFAs [57].

A greater change in the microbiota was observed after insect meal inclusion in broiler diets. On the one hand, low doses of insect meal generally produced a decrease in pathogenic species and an increase in SCFA-producing bacteria [29,58]. On the other hand, high doses of insect meal can increase harmful bacteria with mucolytic activity and decrease SCFA-producing bacteria, with a detrimental effect on gut health [11,17,35]. This discrepancy among the present work and the previous studies could be due to the form in which insects were administered, as live larvae are less rich in nutrients and chitin that insect meals (as mentioned above for mucin expression). Moreover, a potential effect of larval microbiota in modulating the host microbiota can also be hypothesized. A paucity of studies is available on the characterization of HI or TM microbiota, but the results are extremely variable. In fact, the microbiota of insects is influenced by the composition of rearing substrates, which are far from standardized [59,60,61].

## 5. Conclusions

In conclusion, this is the first study evaluating the effect of low doses of HI and TM live larvae as environmental enrichment on the mucin composition, local immune response and microbiota in broiler chickens. The results suggest that insect live larvae do not impair mucin composition or local immune response, and can produce a slight improvement in the caecal microbiota by enhancing a minor fraction of SCFA-producing bacteria. Further studies should be performed to better investigate the potential effects of insect microbiota on the host microbiota.

## Figures and Tables

**Table 1:** Nutrient composition of the commercial feed used in the trial.

	Starter (4–11 Day)	Grower-Finisher (12–38 Day)
Crude protein (%)	22.40	20.00
Ether extract (%)	4.90	5.90
Crude fiber (%)	2.75	2.65
Ash (%)	5.00	3.90
Methionine (%)	0.50	0.47
Lysine (%)	1.20	1.05
Calcium (%)	0.70	0.45
Phosphorus (%)	0.64	0.48
Sodium (%)	0.10	0.10

**Table 2:** Oligonucleotide primers used for rt-qPCR of chicken cytokines.

Type	RNA Target	Primer Sequence	GenBank Accession No.
Reference gene	B-actina	F:5′-GAGAAATTGTGCGTGACATCA-3′ R:5′-CCTGAACCTCTCATTGCCA-3′	L08165.1
	GAPDH	F:5′-GGTGGTGCTAAGCGTGTTAT-3′ R:5′-ACCTCTGTCATCTCTCCACA-3′	K01458
Target gene	TNF-α	F:5′-CCCATCTGCACCACCTTCAT-3′ R:5′-CATCTGAACTGGGCGGTCAT-3′	AY765397.1
	IL-6	F:5′-CAAGGTGACGGAGGAGGAC-3′ R:5′-GGTAGGTCTGAAAGGCGAACA-3′	AJ309540
	INF-γ	F:5′-AGCTGACGGTGGACCTATTATT-3′ R:5′-GGCTTTGCGCTGGATTC-3′	Y07922.1
	IL-2	F:5′-TCTGGGACCACTGTATGCTCT-3′ R:5′-ACACCAGTGGGAAACAGTATCA-3′	AF000631
	IL-4	F:5′-CTTCCTCAACATGCGTCAGC-3′ R:5′-TGAAGTAGTGTTGCCTGCTGC-3′	AJ621735
	MUC-2	F: 5′-ACTCCTCCTTTGTATGCGTGA-3′ R: 5′-GTTAACGCTGCATTCAACCTT-3′	NM.001318434.1

GAPDH: glyceraldehyde-3-phosphate; IFN: interferon; IL: interleukin; MUC: mucin; TNF: tumour necrosis factor; F: forward primer; R: reverse primer.

**Table 3:** Mucin histochemical quantification in the small intestine of the broiler chickens.

	Diet (D)			Intestinal Segment (I)			*p*-Value		
	C	HI	TM	DU	JE	I	D	I	D × I
Neutral mucins, mean (SD)	3.154 (1.18)	3.155 (1.07)	3.563 (1.39)	2.162 ^a^ (0.63)	3.634 ^b^ (0.88)	4.077 ^b^ (1.16)	0.194	<0.001	0.439
Sialomucins, mean (SD)	2.752 (0.85)	2.975 (1.43)	3.345 (2.28)	1.957 ^a^ (0.60)	3.392 ^b^ (1.20)	3.722 ^b^ (2.13)	0.659	<0.001	0.922
Sulfomucins, mean (SD)	3.481 (1.58)	3.302 (1.30)	3.897 (2.04)	2.234 ^a^ (0.91)	4.078 ^b^ (1.79)	4.367 ^b^ (1.32)	0.544	<0.001	0.293
Total mucins, mean (SD)	9.387 (3.19)	9.432 (3.12)	10.805 (4.42)	6.353 ^a^ (1.49)	11.105 ^b^ (2.65)	12.166 ^b^ (3.44)	0.217	<0.001	0.697

C: control; HI: *Hermetia illucens*; TM: *Tenebrio molitor*; DU: duodenum; JE: jejunum; I: ileum. Means with different superscript letters (a and b) within the same column per fixed effect (diet, intestinal segment) differ significantly (*p* < 0.05).

**Table 4:** Relative mRNA expression of gut cytokines and MUC-2 in jejunal tissue of broilers chickens.

	Diet			*p*-Value
	C	HI	TM	
IL-2, mean (SD)	1.879 ^a^ (0.02)	2.417 ^a^ (0.16)	0.568 ^b^ (0.17)	0.044
IL-4, mean (SD)	2.215 (0.28)	1.199 (0.43)	1.805 (0.004)	0.961
INF-γ, mean (SD)	1.259 (0.40)	0.953 (0.45)	1.477 (0.21)	0.860
TNF-α, mean (SD)	1.057 (0.06)	0.639 (0.51)	1.039 (0.66)	0.125
IL-6, mean (SD)	1.865 (0.44)	0.420 (0.11)	1.208 (0.45)	0.146
MUC-2, mean (SD)	1.221 (0.33)	1.518 (0.52)	1.577 (0.37)	0.444

IL: interleukin; HI: *Hermetia illucens*; INF: interferon; MUC: mucin; TM: *Tenebrio molitor*; TNF: tumor necrosis factor. Reference genes (B-actin and GAPDH) were used for normalization of the real-time PCR. Means with different superscript letters (a and b) within the same column per fixed effect (diet, intestinal segment) differ significantly (*p* < 0.05).

## Data Availability

The datasets analysed in the present study are available from the corresponding author on reasonable request. The raw reads data were deposited in the Sequence Read Archive of NCBI (accession number PRJNA761933).

## Supplementary Materials

The following are available online at https://www.mdpi.com/article/10.3390/ani11102819/s1, Script S1: R Script Using for Mucin and rt-qPCR Data Analysis, Script S2: QIIME Script for Microbiota Data Analysis.
