# Supplementary material for: Effect of Insect Live Larvae as Environmental Enrichment on Poultry Gut Health: Gut Mucin Composition, Microbiota and Local Immune Response Evaluation
Source: Animals (Basel). 2021 Sep 27;11(10):2819. doi: 10.3390/ani11102819 (PMC8532707; doi:10.3390/ani11102819)
Supplement: Supplementary file 1 [file animals-11-02819-s001.zip › animals-1380202-supplementary.pdf]

Supplementary Materials

Effect of Insect Live Larvae as Environmental Enrichment on Poultry Gut Health: Gut Mucin Composition, Microbiota and Local Immune Response Evaluation

Elena Colombino [1,*], Ilaria Biasato [2], Ilario Ferrocino [2], Sara Bellezza Oddon [2], Christian Caimi [2], Marta Gariglio [1], Sihem Dabbou [3], Marta Caramori [1], Elena Battisti [1], Stefania Zanet [1], Ezio Ferroglio [1], Luca Cocolin [2], Laura Gasco [2], Achille Schiavone [1] and Maria Teresa Capucchio [1,4]

[1] Department of Veterinary Sciences, University of Turin, 10095 Grugliasco, Turin, Italy; marta.gariglio@unito.it (M.G.); marta.caramori@edu.unito.it (M.C.); elena.battisti@unito.it (E.B.); stefania.zanet@unito.it (S.Z.); ezio.ferroglio@unito.it (E.F.); achille.schiavone@unito.it (A.S.); mariateresa.capucchio@unito.it (M.T.C.)
[2] Department of Agricultural, Forestry and Food Sciences, University of Turin, 10095 Grugliasco, Turin, Italy; ilaria.biasato@unito.it (I.B.); ilario.ferrocino@unito.it (I.F.); sara.bellezzaoddon@unito.it (S.B.O.); christian.caimi@unito.it (C.C.); lucasimone.cocolin@unito.it (L.C.); laura.gasco@unito.it (L.G.)
[3] Center Agriculture Food Environment (C3A), University of Trento, Via E. Mach 1, 38010 San Michele all'Adige, Trentino, Italy; sihem.dabbou@unitn.it
[4] Institute of Sciences of Food Production, CNR, 10095 Grugliasco, Turin, Italy
* Correspondence: elena.colombino@unito.it

1. R Script Using for Mucin and rt-qPCR Data Analysis.

```
library(walrus)
library(readxl)
base <- read_excel("//mac/Home/Desktop/base.xlsx")
View(base)
View(base)
####MUCIN####
###ALCIAN####
shapiro.test(base$a)
ranova(base,
        dep = 'a',
        factors = c('di', 'seg'),
        ph = TRUE)
tapply(base$a, base$di, summary)
tapply(base$a, base_r$seg, summary)
tapply(base$a, base_r$di, sd)
tapply(base$a, base_r$seg, sd)
####PAS###
shapiro.test(base$p)
ranova(base,
        dep = 'p',
        factors = c('di', 'seg'),
        ph = TRUE)
tapply(base$p, base$di, summary)
tapply(base$p, base_r$seg, summary)
tapply(base$p, base_r$di, sd)
tapply(base$p, base_r$seg, sd)
####HID####
shapiro.test(base$h)
ranova(base,
        dep = 'h',
        factors = c('di', 'seg'),
```

```
           ph = TRUE)
tapply(base$h, base$di, summary)
tapply(base$h, base_r$seg, summary)
tapply(base$h, base_r$di, sd)
tapply(base$h, base_r$seg, sd)
####TOTAL MUCIN###
shapiro.test(base$muc_tot)
ranova(base,
           dep = 'muc_tot',
           factors = c('di', 'seg'),
           ph = TRUE)
tapply(base$muc_tot, base$di, summary)
tapply(base$muc_tot, base_r$seg, summary)
tapply(base$muc_tot, base_r$di, sd)
tapply(base$muc_tot, base_r$seg, sd)
###qPCR###
shapiro.test(base1$muc2)
ranova(base1,
           dep = 'muc2',
           factors = c('group'),
           ph = TRUE)
tapply(base1$muc2, base1$group, summary, na.rm=T)
tapply(base1$muc2, base1$group, sd, na.rm=T)
shapiro.test(base1$il_2)
ranova(base1,
           dep = 'il_2',
           factors = c('group'),
           ph = TRUE)
tapply(base1$il_2, base1$group, summary, na.rm=T)
tapply(base1$il_2, base1$group, sd, na.rm=T)
shapiro.test(base1$il_4)
ranova(base1,
           dep = 'il_4',
           factors = c('group'),
           ph = TRUE)
tapply(base1$il_4, base1$group, summary, na.rm=T)
tapply(base1$il_4, base1$group, sd, na.rm=T)
shapiro.test(base1$il_6)
ranova(base1,
           dep = 'il_6',
           factors = c('group'),
           ph = TRUE)
tapply(base1$il_6, base1$group, summary, na.rm=T)
tapply(base1$il_6, base1$group, sd, na.rm=T)
shapiro.test(base1$inf)
ranova(base1,
           dep = 'inf',
           factors = c('group'),
           ph = TRUE)
tapply(base1$inf, base1$group, summary, na.rm=T)
tapply(base1$inf, base1$group, sd, na.rm=T)
shapiro.test(base1$tnf)
ranova(base1,
          dep = 'tnf',
          factors = c('group'),
```

```
    ph = TRUE)
tapply(base1$tnf, base1$group, summary, na.rm=T)
tapply(base1$tnf, base1$group, sd, na.rm=T)
```

2. QIIME Script for Microbiota Data Analysis

```
flash "$x"_R1_001.fastq "$x"_R2_001.fastq -d $x -o $x

multiple_split_libraries_fastq.py -i join/ -o split/ --demultiplexing_method sam-pleid_by_file -- include_input_dir_path --remove_filepath_in_name --parameter_fp phred_quality_threshold 19 -- barcode_type 'not-barcoded' --store_qual_scores

pick_otus.py -i seqs.fna -o picked_otus -s 0.97 -c 0.8

pick_rep_set.py -i picked_otus/seqs_otus.txt -f split/seqs.fna -o rep_set.fna -m most_abundant

assign_taxonomy.py -i rep_set.fna -r gg_13_8_otus/taxonomy/97_otu_taxonomy_ge-nus.txt -o taxonomy -m rdp --rdp_max_memory 64000

align_seqs.py -i rep_set.fna -o pynast_aligned

make_otu_table.py -i picked_otus/seqs_otus.txt -e pynast_aligned/rep_set_fail-ures.fasta -t taxonomy/rep_set_tax_assignments.txt -o otu_table.biom

filter_otus_from_otu_table.py -i otu_table.biom -o otu_table_no_singletons.biom -n 2

filter_alignment.py -i pynast_aligned/rep_set_aligned.fasta -o filtered_alignment

make_phylogeny.py -i filtered_alignment/rep_set_aligned_pfiltered.fasta -o rep_phylo.tre

alpha_diversity.py -i otu_table_.biom -m chao1,shannon,observed_spe-cies,goods_coverage -o adiv.txt

beta_diversity_through_plots.py -i otu_table_.biom -m map.txt -t rep_phylo.tre -o beta_div -e 12.337
```
